# Negative Pressure Wound Therapy in Spinal Surgery

**DOI:** 10.3390/bioengineering9110614

**Published:** 2022-10-26

**Authors:** Alexandra Jeanne White, Ronit Gilad, Soriaya Motivala, Brian Fiani, Jonathan Rasouli

**Affiliations:** 1 Cleveland Clinic Lerner College of Medicine of Case Western Reserve University, Cleveland, OH 44195, USA; 2 Northwell Health—Staten Island University Hospital, Staten Island, NY 10301, USA; 3 Department of Neurosurgery, Weill Cornell Medical Center/New York Presbyterian Hospital, New York, NY 10065, USA

**Keywords:** spine surgery, surgical site infection, wound management, wound dressing, infection prevention, wound healing

## Abstract

Negative pressure wound therapy (NPWT) has demonstrated promise in the management of surgical site infections as well as assisting in surgical wound healing. In this manuscript, we describe the mechanisms and applications of NPWT for surgical wounds and existing evidence for NPWT in cardiac, plastic, and general surgery, followed by a discussion of the emerging evidence base for NPWT in spinal surgery. We also discuss the different applications of NPWT for open wounds and closed incisions, and the promise of newer closed-incision NPWT (ciNPWT) devices. There is nominal but promising prospective evidence on NPWT’s efficacy in select at-risk populations for post-operative wound complications after spinal surgery. As there is currently a paucity of robust clinical evidence on its efficacy, rigorous randomized prospective clinical trials are needed.

## 1. Introduction

Negative pressure wound therapy (NPWT) has become an increasingly popular treatment for the prevention of wound complications and to manage infected and/or chronic wounds healing by secondary intention [1]. Given the significant morbidity and cost associated with surgical site infections (SSIs) in patients undergoing spine surgery, high prevalence of comorbidities that impair wound healing (diabetes, obesity, tobacco smoking), and unique healing challenges inherent to posterior incisions and spinal instrumentation, NPWT holds promise as a potential surgical infection prophylaxis strategy [2]. The goal of this review is to provide an introduction for spine surgeons as to the evidence base for the use of NPWT both in surgery overall and spine surgery specifically, the safety of NPWT, and applications of NPWT for SSI management, SSI prevention, and wound healing. The authors hope that this will provide a comprehensive introduction to the rationale for NPWT use, potential applications, and gaps in NPWT research. This review will describe the mechanisms and evidence base for NPWT as an intervention for both the management and prevention of SSI and other wound complications such as wound dehiscence. It will then review the epidemiology and risk factors for SSI in spine surgery and discuss the existing evidence for NPWT in spine surgery. 

## 2. Mechanisms and Evidence for NPWT

NPWT is a closed system that applies suction, i.e., negative pressure, to the surface of a wound. This system consists of an open-pore foam that fills the wound or overlies a closed incision, an airtight adhesive drape, and a vacuum pump that creates and maintains negative pressure [3]. The use of foam allows for uniform distribution of negative pressure across the wound surface, as well as the egress of fluids through the foam [3]. NPWT was originally intended to improve serum drainage and ensure adherence to skin flaps but was later found to improve infection control as well [3]. NPWT is applied by placing a gauze or open-cell foam dressing over the wound and sealing it with an occlusive drape, then connecting it to intermittent or continuous suction to a vacuum pump and liquid waste collector [4]. An amount of 125 mm Hg is the optimal negative pressure, as it has been shown to promote sufficient formation of granulation tissues without compromising perfusion to the wound [5]. The vacuum seal enables stability in the wound environment, keeps it moist and warm, and prevents bacterial colonization of the wound [6]. In a porcine model, NPWT reduced bacterial count inside wounds compared to conventional gauze dressing [7]. Vacuum pumps, such as Vacuum Assisted Closure (V.A.C.^®^) [6] (3M^TM^, St. Paul, MN, USA) are a commonly used device, although additional options are becoming rapidly available (Table 1). 

Putative mechanisms of NPWT include augmentation of local blood flow and promotion of granulation tissue formation, as well as reduction in bacterial contamination, edema, and exudate [1,8,9]. Importantly, NPWT preserves a moist environment in the wound, which is key for wound healing [9]. Negative pressure suction removes edema, blood, and serous fluid (all of which contain inflammatory mediators) from the wound and opens capillaries in close proximity to the wound edge [9,10,11]. NPWT exerts contractile forces that promote approximation of the wound edges and reduce stretch on the edges of the wound [3]. Microdeformation caused by adherence of the foam to the wound surface may also promote wound healing and facilitate a favorable wound microenvironment, preventing the development of chronic wounds [3]. Specifically, these mechanical microdeformation forces lead to inhibition of apoptosis, changes in cell signaling and gene expression, and promotion of cellular proliferation [6]. Cell signaling changes include changes in expression of vascular endothelial growth factor, interleukin-6 and interleukin-8 [11]. In addition, NPWT devices allow for more prolonged periods of time between dressing changes, which can cause a great deal of pain and discomfort to patients. Nonetheless, studies of the impact of NPWT on patient quality of life (QOL) have been inconclusive, with a 2016 systematic review reporting some evidence that NPWT is associated with decreased QOL in the first week compared to standard wound care, but that QOL then improves [12]. The use of NPWT systems is also associated with increased cost given the expense of these patented devices compared to conventional gauze dressing.

NPWT can be applied to open surgical or traumatic wounds, closed incisions in high-risk patients, chronic wounds, and skin grafts [3]. They can be used as a temporary measure in preparation for delayed closure or the placement of a skin flap. In addition, instillation of sterile water, saline, antibiotics, or antiseptics into the wound while NPWT is applied is an emerging therapeutic advance [13]. Adherence to treatment at home can also be monitoring in newer devices (Table 1). NPWT has been employed in a variety of wounds: open abdominal wounds, open fractures, burn wounds, pressure ulcers, post-traumatic wounds, diabetic foot ulcers, split-thickness skin grafts, sternal wounds, and in patients with obesity after clean incisions [1]. Among the few contraindications to NPWT are untreated fistulas, untreated osteomyelitis, the presence of a malignant tumor in the wound, the presence of exposed vasculature or nerves, and the presence of necrotic tissue with eschar [9]. A 2019 meta-analysis of randomized controlled trials of NPWT for SSI prevention demonstrated that NPWT is associated with a 40% lower risk of SSI compared to standard wound care [14]. However, none of these studies were conducted in the realm of spinal surgery.

The use of closed-incision NPWT (ciNPWT) is gaining popularity across surgical subspecialties. Preclinical studies identified increased wound perfusion and increased mechanical properties and tensile strength when NPWT was applied to closed surgical wounds [15,16,17]. A 2017 systematic review found that ciNPWT in a variety of surgical subspecialties was associated with reduced wound complications, wound dehiscence, surgical site infections, hematoma/seroma formation and incisional drainage [8]. In addition to providing the overall perfusion and tissue healing benefits of NPWT, ciNPWT likely reduces incision line tension, decreases edema, and ensures an airtight seal [8]. The authors recommend the use of ciNPWT in areas where infection can cause high morbidity, e.g., median sternotomy, groin area vascular surgery, or surgeries with insertion of hardware and in patient populations with risk factors for SSI (diabetes mellitus, ASA score > 3, older age, obesity, current smoking, use of corticosteroids) [8]. In light of this, spine surgery patients are appropriate candidates for ciNPWT given the serious morbidity of spine SSIs and the extensive use of instrumentation in spinal surgery. 

Prevena^TM^ Incision Management System (3M^TM^, Minneapolis, MN, USA) is a frequently used NPWT device specifically designed for ciNPWT [2] (Table 1). Unlike intra-wound NPWT therapies, Prevena ^TM^ is an external, incisional wound vacuum system that is designed to be portable and easily transportable for ambulatory patients. It consists of a vacuum therapy suction unit that connects to a suction canister, a precut open-cell foam that is placed over the incision, a plated non-adherent layer surrounding the foam, and an adhesive drape. The dressing is impregnated with ionic silver to prevent the growth of bacteria [2]. Pico System (Smith + Nephew, London, UK) is another, canister-free ciNPWT that can be in place for as long as 7 days and allows for safe discharge of patients with the dressing in place [2] (Table 1).

A 2015 meta-analysis demonstrated significantly decreased incidence of SSIs in ciNPWT compared to standard wound care (29.4% relative reduction), with an additional reduction in overall rates of dehiscence [18]. This was further supported by a 2016 meta-analysis which also revealed a significant reduction in wound infections with the use of ciNPWT (RR 0.54, 95% CI 0.33–0.89) [19]. However, a 2019 Cochrane review of NPWT for surgical wound healing by primary intention (i.e., with a closed incision) revealed only low-quality evidence that NPWT may reduce the incidence of SSI, suggesting that further study is needed [1].

Sternotomy wounds are perhaps the closest comparison to spine wounds, and there is robust evidence of VAC and Prevena usage in sternotomy incision management [20]. The use of NPWT is particularly relevant in cardiac surgery given the life-threatening consequences of a sternal SSI. A 2022 meta-analysis suggested that NPWT may prevent postoperative sternal SSI after cardiac surgery, although the quality of the evidence in the studies that were identified was fair and poor [21]. On the other hand, a 2013 randomized controlled trial of Prevena wound dressings vs. conventional wound dressings in obese patients undergoing cardiac surgery with closed incisions demonstrated a significantly reduced incidence of postoperative SSI in the Prevena group (OR 4.75, 95% CI 1.23–16.94) [22]. The use of Prevena dressings also significantly reduced the incidence of SSI in patients with deep sternal wound infection that required muscle flap reconstruction [23].

In addition, the use of NPWT in orthopedic surgery may also shed light on NPWT and spine surgery. ciNPWT has been demonstrated to be safe and effective in traumatic fractures, as well as in preventing prosthetic joint infection after total joint arthroplasty [24]. A 2018 meta-analysis of the use of NPWT for open fractures also demonstrated that it is associated with lower SSI, faster wound healing, and reduced length of stay [25]. 

There is emerging evidence for the cost-effectiveness of NPWT compared to standard dressings in non-spinal surgery. For example, the randomized INVIPS trial of NPWT after open inguinal vascular surgery demonstrated that while NPWT was more costly, the use of NPWT did not increase procedure-related costs and resulted in a significantly reduced SSI incidence, thus suggesting that NPWT in this setting is a cost-effective intervention [26]. On the other hand, NPWT was not shown to be a cost-effective intervention in adults with severe open lower limb fractures, due to higher costs and only marginally higher QALYs in the NPWT group [27]. In women undergoing cesarean section, incisional NPWT appeared to be cost saving primarily in women with a BMI ≥ 35 kg/m^2^ [28]. This underscores the importance of rigorous cost-effectiveness studies that specifically evaluate the value of NPWT in spinal surgery. 

## 3. NPWT in Spine

### 3.1. NPWT for Infection Management

The majority of evidence in spine surgery focuses on the use of NPWT for management, not prevention, of SSI, and is retrospective in nature [29]. Postoperative SSI is a major issue in spine surgery. SSIs are second only to pneumonia and urinary tract infection in incidence after spine surgery and are linked to increased cost of treatment [30]. Risk factors include elevated serum glucose, BMI, diabetes, male sex, hypertension, coronary artery disease, American Society of Anesthesiology (ASA) score > 3 and prologed use of corticosteroids [30]. Risk of SSI is higher in deformity surgery, fusion of higher numbers of level and surgery in the setting of trauma or preexisting infection, as well as with the use of instrumentation or allograft given the introduction of foreign material into the wound. Thus, there is a strong need for SSI management approaches, such as NPWT, in spine surgery. 

In these cases, NPWT serves as an adjunct to careful debridement in the operating room and antibiotic therapy and is applied over the debrided wound [11]. NPWT dressing changes can be conducted in the operating room to minimize the risk of wound contamination [11]. An early retrospective study demonstrated the safety of VAC use in complex spine wound management in twenty patients after spinal fusion [31]. A 2006 retrospective case series of fifteen patients was one of the first to demonstrate that VAC therapy was an option for management of complex postsurgical spine wounds, allowing ultimately for closure of all the wounds (thirteen by suturing, two by muscle flap) [32]. A more recent series of 21 patients who underwent posterior lumbar fusion with instrumentation and developed SSI managed with NPWT, all patients retained instrumentation after successful treatment with NPWT [33].

NPWT has also been successfully implemented in pediatric spine surgery [34,35]. This was initially demonstrated in a 2007 case series of six patients with deep wound infection following instrumented fusion for neuromuscular scoliosis and a retrospective review of 249 patients, 11 of whom developed a wound infection and underwent VAC placement [36,37]. Similarly, Canavese et al. reported in 2008 that they were able to retain instrumentation in a series of 14 pediatric patients who developed wound infections after fusion for neuromuscular scoliosis, demonstrating the efficacy of this system in larger wounds with 10-level fusions [34]. However, they note that patients in their series still required multiple procedures prior to wound closure [34]. This group later reported that NPWT is not contraindicated in patients treated with hybrid constructs with sublaminar bands [35]. These findings are especially relevant given the higher rates of postoperative SSI in patients with neuromuscular scoliosis compared to idiopathic scoliosis [35].

Although the majority of studies do not report complications associated with use of the VAC system in management of postoperative wound infections, one study reported a postoperative death due to hemorrhage that was apparently exacerbated by placement of the wound VAC. This hemorrhage and subsequent death occurred in the setting of high intraoperative blood loss and refusal of transfusion on religious grounds [38]. On the other hand, NPWT has more recently been described as a management tool for symptomatic epidural hematoma after spine surgery [39]. Foxx et al. published four cases (three cervical, one lumbar) in 2014, in all of which they demonstrated the successful use of NPWT In high-risk patients presenting with epidural hematoma [39]. In one of these cases, the patient experienced uncontrollable intraoperative hemorrhage. Due to concern about the risk of infection, all patients received antibiotic prophylaxis [39]. The safety and feasibility of NPWT has also been demonstrated in a sample of 16 patients with complex craniofacial and cervical wounds, which further supports the use of NPWT in cervical spine surgery [40]. The authors recommend that the area be thoroughly shaved and cleaned, as hair can interfere with the seal created by the dressing [40].

In 2017, Watt et al. discussed the advantages of NPWT in managing deep SSI after surgery specifically when it comes to the management of dead space [41]. Exposure, resection of posterior elements for decompression, and restoration of lordosis make spine surgery especially prone to the formation of dead space, which can lead to the formation of hematomas and ultimately areas for the spread of infection [41]. The authors posit that vacuum dressings decrease the potential space, remove excess fluid and maintain a lack of tension on surrounding tissues [41]. Importantly, VAC therapy has been associated with preservation of hardware after SSI in numerous studies, thus reducing cost and morbidity associated with hardware removal [34,42,43,44].

According to a retrospective study by Ploumis et al., recurrence of wound infection after VAC treatment is not related to depth of infection, presence of instrumentation, age, or other comorbidities [43]. It may be related to the presence of MRSA in the wound or multibacterial infections. The use of NPWT in SSI management after spine surgery can also minimize the need for flap closure, which is associated with significant morbidity [45]. Even when tissue flaps are required, the VAC stimulates granulation tissue formation [3], which in turn facilitates flap placement.

On the other hand, Yuan et al. demonstrated that deep wound infection management with wound VACs, compared to continuous irrigation suction systems (CSIS), were associated with increased cost and hospital stay [46]. The advantages of irrigation include potential reduction in infection rates and removal of exudates and infectious material from the wound bed [47]. On the contrary, Shi et al. demonstrated in a retrospective study of a prospective database that the use of wound VACs combined with CSIS, compared to CSIS alone, in managing early deep SSI after posterior lumbar fusion with instrumentation, resulted in reduced hospital length of stay, duration of antibiotic use, number of dressing changes, and number of debridements [48]. One group reported success in SSI management after spine surgery with the use of a modified NPWT device that used 3000 mL of saline lavage, which was replaced daily [47]. Similarly, a case report describes the use of NPWT with instillation of saline for a patient who developed SSI, dehiscence and a chronic wound after an L4-5 transforaminal lumbar interbody fusion (TLIF), which required multiple revision surgeries [49]. NPWT alone was not effective for this patient, suggesting that NPWT with instillation can be a form of escalation therapy for nonhealing wounds [49]. 

A pooled analysis in a 2022 systematic review demonstrated that VAC therapy resulted in an absolute risk reduction of 16.6% and relative risk reduction of 40.4% for implant removal in SSI after posterior instrumented fusion, thus leading to a number needed to treat of 6.0 [50].

Traditionally, when managing SSI, wound VACs are placed directly over the open wound. A 2020 retrospective study compared this technique to placement of the VAC sponge over reapproximated paraspinal muscles and found that suprafascial VAC was associated with shorter duration of therapy, reduced risk of a retained sponge fragment, and fewer subsequent procedures [51].

Nonetheless, recurrence of SSI after debridement and VAC therapy has been reported. In a retrospective study of 178 patients undergoing spine surgery who developed an SSI, 14 experienced at least one SSI recurrence [40]. In this population, recurrence was not associated with obesity, diabetes, MRSA infection, fusion surgery or duration of VAC treatment [52]. There is also no current consensus on the appropriate duration of VAC therapy in spine surgery patients. 

#### Safety of NPWT over Exposed Spinal Dura

A key study demonstrated the safety of NPWT in patients with deep SSI and durotomy or dural exposure, as NPWT did not result in CSF-related complications [53]. Some sources recommend reduced pressure in wounds with exposed dura (e.g., 50 mm Hg vs. 125 mm Hg) [42]. This is echoed by a case report describing the safe application of NPWT to the dura in a patient with a severe scalp burn [54].

### 3.2. NPWT for Infection Prophylaxis

A smaller number of studies have examined the application of NPWT in spine surgery for the prevention of infection. In 2014, NPWT was reported to significantly reduce the incidence of postoperative wound infection (10.63% vs. 14.91%), wound dehiscence (6.28% vs. 12.28%), and reoperation rates in a retrospective cohort study at Duke University [55]. In this population, all patients undergoing long-segment spine fusions for idiopathic scoliosis received closed-incision NPWT for 3 days postoperatively [55]. ciNPWT (PICO system) was also found to be associated with reduced development of postoperative seroma, as well as time and materials spent on wound care, in a 2016 prospective study of 20 patients [56]. A retrospective proof of concept study demonstrated that use of an incisional Prevena VAC after primary closure compared to standard care in a high-risk patient population prone to SSI after spine surgery (posterior open surgery across the cervicothoracic junction; thoracic kyphosis due to metastatic disease; high-energy trauma; or multilevel revision reconstructive surgery) reduced the incidence of SSI by 50% (10% vs. 21%) [57]. This was despite the VAC group having more risk factors for SSI than the standard wound care group, such as malnourishment, longer operative time, and increased ICU admission [57]. However, this finding was not statistically significant, likely owing to the small sample size [57]. A 2021 retrospective matched cohort study demonstrated that Prevena NPWT is associated with reduced incidence of SSI when controlling for sex and BMI in patients undergoing posterior spinal fusions [58]. However, the control cohort in this study had an unusually high incidence of SSI (7.1% or 3 out of 42 patients) [58]. A 2021 retrospective, non-randomized study of a small patient population showed that the use of NPWT in patients with metastatic spine disease and instability or accelerated neurological decline resulted in reduced incidence of SSI postoperatively, which is important given the high risk of wound complications in this population [59]. These studies underscore the urgent need for high-quality prospective randomized trials of NPWT in spine surgery. 

However, not all evidence supports the use of NPWT as an infection prevention strategy. Naylor et al. reported that, although NPWT was used selectively in patients felt to be at especially high risk of postoperative wound complications, the incidence of wound dehiscence and SSI was 5.6% in the non-NPWT group and 5.7% in the NPWT group (*p* = 0.98) in a retrospective study of a single surgeon’s experience with NPWT [60].

A recent large-scale (*n* = 274) prospective observational study revealed that ciNPWT at the time of the index surgery was associated with a significant reduction in SSI rates in patients undergoing a wide range of spine procedures, including anterior approaches [61]. The authors recommend the selective use of ciNPWT for instrumented operations and for other higher risk populations, given its higher upfront cost [61]. In a similar vein, a 2021 meta-analysis of prophylactic NPWT in spinal fusion found that NPWT was effective in reducing the incidence of postoperative SSI, but did not have any effect on wound dehiscence, wound complications, and rates of reoperation and hospital readmission [62]. 

Several clinical trials examining the use of incisional NPWT (typically with the use of Prevena) in patients undergoing spine surgery have been reported, but not published. A randomized trial evaluating the prophylactic use of an incisional wound VAC after posterior spine surgery in patients with BMI > 35 (NCT02926924) is actively recruiting patients. 

Wound VACS to manage SSI after spinal surgery can be recommended as long as the patient does not have contraindications to the use of NPWT (malignant tumor in the wound, the presence of exposed vasculature or nerves, and the presence of necrotic tissue with eschar) (Table 2). Wound VACS can be safely applied to a wound with exposed, intact dura without durotomy. In addition, the use of ciNPWT devices have been shown to prevent infection and promote wound healing in select patients at high risk for wound dehiscence (obesity, diabetes mellitus, chronic steroid use, metastatic spine disease) in spinal surgeries that carry a risk of poor wound healing (e.g., posterior open surgery across the cervicothoracic junction; thoracic kyphosis due to metastatic disease; high-energy trauma; or multilevel revision reconstructive surgery) (Table 2). There is no published data on the cost-effectiveness of NPWT use in spinal surgery.

## 4. Conclusions

This review summarizes the existing evidence surrounding the use of NPWT in spine surgery, synthesizing it with an understanding of the mechanisms of NPWT and its applications in other surgical subspecialties. NPWT is a promising tool in managing SSI and has broad applications in spine surgery, given the morbidity and mortality associated with SSI in this population (Table 1). There is greater evidence for the use of NPWT in prevention of SSI compared to the prevention of other wound complications. Putative mechanisms include facilitation of microcapillary perfusion microdeformation forces, as well as protecting the wound from contact with the outside world and thus contamination. Importantly, NPWT appears to allow for the retention of instrumentation after SSI. Moreover, it is safe and effective in management of hematomas and in the presence of exposed dura. ciNPWT (e.g., Prevena) is a promising tool for the prevention of SSI and wound dehiscence in high-risk populations, such as patients with obesity, diabetes mellitus, or metastatic spine disease or in high risk operations, such as deformity correction across multiple vertebral levels or surgery in the setting of trauma. Given the high prevalence of comorbidities such as obesity and diabetes mellitus in patients undergoing spinal surgery, which increase the risk for both SSI and wound complication, the use of NPWT is especially relevant. While early results are promising, there is an urgent need for large-scale prospective randomized controlled trials of ciNPWT vs. standard wound care, as well as cost effectiveness analyses of traditional NPWT and ciNPWT compared to conventional dressings specifically in the setting of spine surgery. Once more evidence is collected, formalized indications for NPWT in spine surgery based on patient characteristics and surgical aspects will likely help reduce morbidity and costs associated with SSI and wound complications in a targeted manner. 

## Figures and Tables

**Table 1:** Currently Available NPWT Devices.

Name	Manufacturer	Uses	Portable?
V.A.C.^®^ Ulta	3M^TM^	NPWT, instillation	No
V.A.C.^®^ Simplicity	3M^TM^	NPWT, ciNPWT	Yes
V.A.C.^®^ Rx4	3M^TM^	NPWT in up to 4 wounds	No
ActiV.A.C.^®^	3M^TM^	NPWT, home care	Yes
ActiV.A.C.^®^ with iOn Progress^TM^	3M^TM^	NPWT with monitoring of home use	Yes
V.A.C.^®^ Via	3M^TM^	NPWT for 7 days	Yes
Pico System	Smith + Nephew	ciNPWT	Yes
Prevena^TM^	3M^TM^	NPWT, ciNPWT	Yes

**Table 2:** Summary of Recommendations for the use of NPWT in Spinal Surgery.

Proposed Use	Recommendation	Level of Evidence
Treatment of deep and superficial SSI	Wound VACs are effective in managing SSI	2A
ciNPWT for infection prophylaxis	Supported by literature in orthopedic and cardiac surgery	2A
Routine use of ciNPWT	Likely not beneficial	3B
Routine use of ciNPWT in “high-risk” patient populations	Potentially beneficial; more studies are needed	2B
Cost-effectiveness	Unknown	-

## Data Availability

Not applicable.
